# Phylogenetic inference from single-cell RNA-seq data

**DOI:** 10.1038/s41598-023-39995-6

**Published:** 2023-08-08

**Authors:** Xuan Liu, Jason I. Griffiths, Isaac Bishara, Jiayi Liu, Andrea H. Bild, Jeffrey T. Chang

**Affiliations:** 1 Department of Integrative Biology & Pharmacology, University of Texas Health Science Center at Houston, 6431 Fannin St, MSB 4.218, Houston, TX 77030 USA; 2 Division of Molecular Pharmacology, Department of Medical Oncology & Clinical Therapeutics, City of Hope, Monrovia, CA USA

**Keywords:** Phylogeny, Tumour heterogeneity

## Abstract

Tumors are comprised of subpopulations of cancer cells that harbor distinct genetic profiles and phenotypes that evolve over time and during treatment. By reconstructing the course of cancer evolution, we can understand the acquisition of the malignant properties that drive tumor progression. Unfortunately, recovering the evolutionary relationships of individual cancer cells linked to their phenotypes remains a difficult challenge. To address this need, we have developed PhylinSic, a method that reconstructs the phylogenetic relationships among cells linked to their gene expression profiles from single cell RNA-sequencing (scRNA-Seq) data. This method calls nucleotide bases using a probabilistic smoothing approach and then estimates a phylogenetic tree using a Bayesian modeling algorithm. We showed that PhylinSic identified evolutionary relationships underpinning drug selection and metastasis and was sensitive enough to identify subclones from genetic drift. We found that breast cancer tumors resistant to chemotherapies harbored multiple genetic lineages that independently acquired high K-Ras and β-catenin, suggesting that therapeutic strategies may need to control multiple lineages to be durable. These results demonstrated that PhylinSic can reconstruct evolution and link the genotypes and phenotypes of cells across monophyletic tumors using scRNA-Seq.

## Introduction

Since it was first documented by Nowell^1^, the cancer cell evolution model where mutation and selective pressures produce cancer cell lineages with heritable malignant traits has gained an increasing amount of support^2,3^. Cancer cell lineages with distinct genotypes, also referred to as subpopulations or subclones, could share common phenotypes^4^, or may exhibit different malignant properties impacting proliferation rates, drug resistance, or metastatic capacity. Therefore, each lineage may require different treatment strategies. To choose a targeted therapy, an understanding of the evolution of individual cancer cells in a tumor will help to reveal the number of cancer lineages that are being treated, and help uncover the traits that are under genetic selection, how they are acquired, and also potential targets to inhibit each lineage.

Early methods to explore tumor genetic diversity applied next generation sequencing of the DNA (genome, exome, or select targets) to bulk tumor samples and used the frequency of mutated reads to infer cancer subclones^5,6^. To uncover the genetic architecture of cancer cells in a tumor, single-cell barcoding strategies have been developed^7–10^. This allowed a greater resolution of evolutionary changes using single-cell DNA sequencing (scDNA-Seq)^11^. However, while these methods could reconstruct the evolutionary histories of cancer cells, they did not reveal the phenotypes of the evolving cancer cells. To study cellular phenotypes, methods have been developed that could infer linkages between scDNA-Seq and scRNA-Seq (single-cell RNA sequencing) data in samples where profiling was performed in parallel but on different cells^12^. Although single-cell multi-omic protocols to sequence DNA and RNA from the same cell have been developed, this strategy has remained technically challenging to implement^13–15^.

A low-resolution approach to link cellular genotype and phenotype within a tumor was to find subclones from only scRNA-Seq data. A frequently used method was to predict copy number variation from gene expression measurements, and then to perform hierarchical clustering on the copy numbers^16,17^. Although the resultant dendrogram resembled a phylogeny, it did not reflect an underlying model of evolution and relied on the assumption that regions of upregulated gene expression reflected changes in genetic copy number and not, for example, transcriptional activation. Further, interpreting dendrogram branching points as evolutionary events in the history of the growth of the tumor required the implicit and unsupported assumption that the metric of distance between copy numbers reflected the time needed to acquire the copy number alterations. Thus, without evolutionary models of (i) a molecular clock of mutational events, (ii) nucleotide substitution probabilities, or (iii) the branching process, it was difficult to interpret the history and divergence of groupings of the cancer cell subclones as evolutionary events.

One approach to address these deficiencies used phylogenies inferred from single nucleotide variants seen in the scRNA-Seq data^18^, but encountered difficulties due to the low coverage and high drop-out rates seen in scRNA-Seq data^19^. As a consequence, few cells were studied, cancer cells were difficult to distinguish from normal cells, and distinct clades of cancer cells were hard to identify. Other tools have been developed to cluster cells based on mutations called from their scRNA-Seq profiles into subclones^20^, a dendrogram structure^21^, or a clonal tree incorporating longitudinal time points^22^. The algorithm most similar to ours was the second method, DENDRO, whose authors explicitly recommended against applying it to sequencing methods that did not profile full length transcripts, including 10X, currently the most common scRNA-Seq platform, due to lack of variants and low read coverage. Thus, strategies that could overcome the challenges from scRNA-Seq data were still needed.

One strategy to circumvent the quality issues in scRNA-Seq was to predict subclonal structure from bulk DNA sequencing on the same samples, and then assign cells from scRNA-Seq to those subclones, an easier task than generating the phylogeny de novo^23–25^. However, a major drawback was the requirement of bulk sequencing of the same samples, which was not always possible or available. Further, it did not circumvent the need to have accurate mutation calls in the scRNA-Seq data, which remained a challenge.

We also note that a multitude of algorithms now exist to model genetic relationships amongst cells profiled by single cell DNA sequencing^26–34^. Here, a number of approaches have been developed that could integrate errors from sequencing, amplification, allelic drop out, and cell doublets into the tree generation algorithm, including phylogenetic-aware base imputation in the BEAM method^35^. For a review of these strategies, see^36^. However, while largely successful in improving performance, compared to RNA, DNA-sequencing has overall broader coverage across the targeted regions, transcriptomes, or genomes; and was not confounded by widely varying levels of expression of genes that resulted in the low gene numbers and uneven coverage across genes that was seen in scRNA-Seq data. Coverage was also uneven within a transcript, as the sequencing in the most commonly used protocols, including the 10X 3′ gene expression solution, was biased toward the 3′ end, further limiting the profiling of the mutations. Thus, while in principle the same algorithms could be applied to mutations from the DNA and RNA, the source and nature of the errors differed significantly, and methods needed to be developed that can address the challenges seen in RNA.

To infer evolutionary relationships (phylogenies) of single cancer cells from noisy real-world scRNA-Seq data, we have developed a method, PhylinSic (Phylogeny in Single cells, created in a pandemic and pronounced “feeling sick”). It addresses the challenge of constructing an evolutionary model from low coverage scRNA-Seq data by calling nucleotide bases using information borrowed from genetically similar cells, and then applies a Bayesian phylogenetic inference algorithm, BEAST2, to model single cell evolutionary history using the imputed base calls^37^. To verify that PhylinSic accurately revealed evolutionary relationships in real biological scenarios, we evaluated its performance on scRNA-Seq datasets collected from a range of cell culture and patient samples and across different cancer types and disease stages. We evaluated PhylinSic using a data set of ER+ breast cancer cells where experimental evolution yielded fluorescently labeled drug-resistant and -sensitive isogenic lineages that could be distinguished in the scRNA sequencing data. We found the method to be robust to the inherent noise in scRNA-Seq data and able to reconstruct cancer cell phylogenetic relationships and uncover how phylogeny was reflected in cell phenotypes. We applied PhylinSic to investigate drug resistance in breast cancer and multiple myeloma tumors, showing its applicability across biological contexts. In all data sets, we found evidence of genetic evolution across disease progression, as well as evidence for convergent evolution where multiple lineages evolved toward a common mechanism of resistance. PhylinSic shares information between genotypically similar cells to robustly reconstruct phylogenetic relationships from inherently noisy scRNA-Seq data and has proven to be applicable to data collected from patient tumors.

## Results

### Modeling phylogenies from scRNA-Seq data

**Figure 1:**
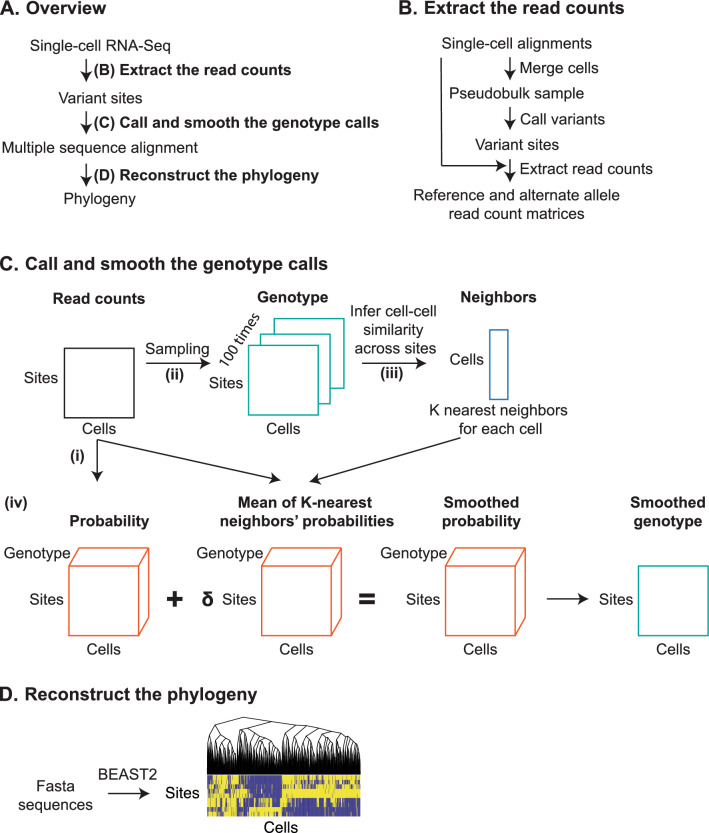
Construction of phylogeny from scRNA-Seq data. (**A**) Generating a phylogeny from scRNA-Seq alignments consists of three major steps: (1) extracting the read counts, (2) calling and smoothing the genotypes, and (3) reconstructing the phylogeny. (**B**) The read count extraction process: We start with the alignments of cells from a single-cell RNA-Seq experiment. To extract matrices of read counts, we identify sites of interest by merging the alignments in a pseudobulk sample and calling variants. Then, at each of the variant sites, in each individual cell, we count the number of reads with the reference and alternate alleles. (**C**) The genotype calling and smoothing processes: We start from matrices of read counts of reference and alternate alleles seen across sites (rows) in single cells (columns). (i) Given the number of reads, we assigned a probability of a (R)eference, (A)lternate, or (H)eterozygous genotype by integrating over a beta-binomial density function. (ii) To compare the genotypes of two cells, we sample 100 genotype profiles by drawing from their probability distributions. (iii) Comparing every pair of cells leads to a pairwise similarity matrix of genetic distance scores. By looking for the highest scores (excluding itself), we find the *K* nearest neighbors for each cell. (iv) With the nearest neighbors, we can smooth the genotype probability of a cell by averaging with the weighted ($\delta$) average probabilities of its neighbors. We call the genotype with the highest probability score. (**D**) Phylogenetic reconstruction: We use BEAST2 to infer the phylogeny and produce a final tree using the max clade credibility method.

We developed an algorithm to estimate the evolutionary relationships among monophyletic cells (e.g., those derived from the same person) profiled with scRNA-Seq technologies. The algorithm had three major steps: (1) identification of variant sites, (2) inference of the nucleotide bases at variant sites for each cell, using smoothing to account for scRNA-seq noise, and then (3) reconstruction of the phylogenetic history using an evolutionary model (Fig. 1A).

The algorithm first identifies sites that vary across the cells and thus might best reveal phylogenetic structure. To do this, we combine the reads for all the cells into a single pseudobulk sample and then called variant sites using a GATK pipeline (Fig. 1B). Next, for each cell, we call a genotype (either reference, alternate, or heterozygous) at each of the variant sites (Fig. 1C). To account for low read depth, genotype calls are smoothed using information from related cells, as described in the “Methods”. We assign reference and alternate bases according to the base seen in the alignments, and if the genotype was heterozygous, we assign an arbitrary surrogate base. Finally, to estimate the phylogeny of the cells, we use BEAST2, a Bayesian phylogenetic inference algorithm (Fig. 1D)^37^.

### Smoothing genotypes enables identification of resistant phenotypes

To verify that genotype smoothing (from Fig. 1C) helps recover more accurate phylogenetic relationships, we used a data set where the CAMA-1 breast cancer cell line was experimentally evolved under 6 months of treatment with ribociclib, a CDK4/6 inhibitor, resulting in a ribociclib resistant cell line (GSE193278)^38^. A sister lineage from the same ancestral population evolved under untreated conditions for the same period and remained ribociclib sensitive. Whole exome sequencing revealed genetic differences between the resistant and parental populations, including nine predicted to be highly deleterious, suggesting that the resistant cells underwent genetic evolution under drug selection^39^. The populations of sensitive and resistant cells were fluorescently labelled allowing them to be distinguished when co-cultured. Cells were grown for 14 days in both mono- and co-culture, allowing us to distinguish biological differences in resistant and sensitive cell genetic profiles from possible technical batch effects of separate culture.

We pooled scRNA-Seq data from all cells, processed the data (as described in the “Methods”), and obtained 523 variant sites and 400 cells (200 resistant and 200 sensitive). We inferred cancer mutation profiles and phylogenetic trees of the cells’ evolutionary relatedness with or without application of our algorithm for smoothing the genotypes. Assuming that ribociclib resistance evolved due to selection for a lineage of cells that harbored genetic changes allowing proliferation under treatment, we expected to observe that resistant and sensitive cells (i) had distinct mutational profiles and (ii) occupied different evolutionary branches of the phylogeny.

**Figure 2:**
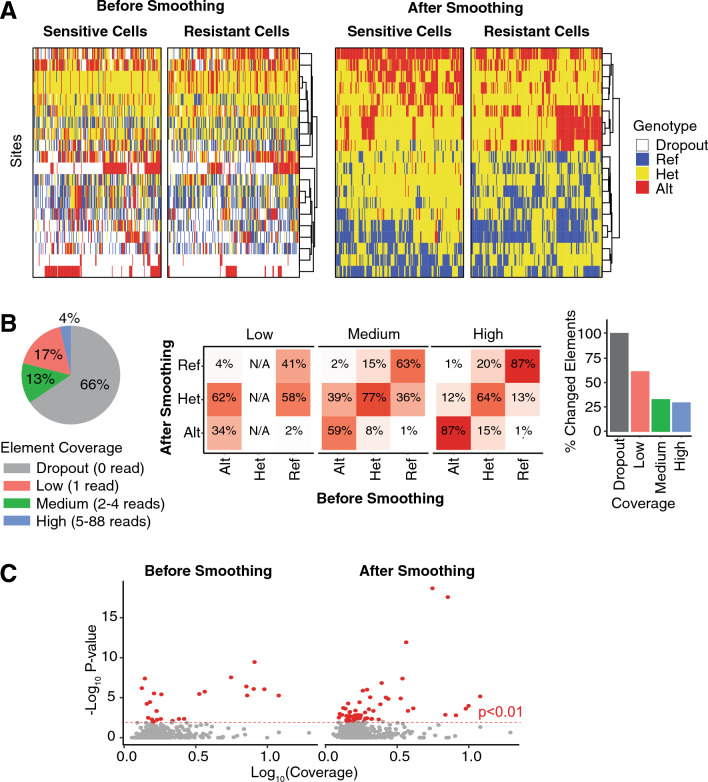
Smoothing improves the distinction of the genotype profile of the resistant and sensitive cells. (**A**) Heatmaps show the genotype profile of the resistant and sensitive cells (columns) for the 20 sites (rows) that are most significantly associated with resistance. The heatmaps on the left show the genotype profiles before smoothing, and the ones on the right show the genotype profiles after smoothing. The genotypes are called as either Reference (blue), Alternate (red), or Heterozygous (yellow). Missing data (drop-out) are shown in white. (**B**) (left panel) The pie chart shows the distribution of the number of reads seen in each element in the site x cell matrix. The fraction of elements with no reads (dropout—the site is not seen in a cell) is shown in grey, the ones with a single read is shown in red, 2–4 reads is green, and at least 5 reads in blue. (middle panel) The tables show how the genotype frequencies changed after smoothing. The elements are discretized into Low (1 read; left table), Medium (2–4 reads; middle table), and High (5+ reads; right table) coverage groups. The columns indicate whether the genotype was Alt(ernate), Het(erozygous), or (Ref)erence before smoothing, and the rows indicate the genotype after smoothing. Each cell in the table indicates the percent of elements that were changed. Each column adds up to 100% (after accounting for rounding artifacts). The Het column in the Low coverage coverage group contains N/A because we cannot call a heterozygous genotype from only 1 read without smoothing. (right panel) The bar plot shows the percent of elements in each coverage group that are changed. (**C**) The scatter plots show the association between the mean coverage (x-axis) of each mutation site (points) and the correlation of its predicted genotype with the resistance phenotype (y-axis) either before (left plot) or after smoothing (right). Sites associated with the resistance phenotype at p < 0.01 are shown in red.

When we compared the mutation profiles generated with and without genotype smoothing, we found that the nucleotide bases called using smoothing better distinguished the sensitive and resistant cells (Fig. 2A, the 20 sites most significantly associated with resistance are shown). Without smoothing and imputation, 30% of the values in the matrix were missing, and the genotypic variation showed no association with the resistance status (adjusted Rand Index = 0, p = 0.44). With smoothing, the genotypes at selected variant sites were significantly associated with the resistance lineage (adjusted Rand Index = 0.3, p = 0.001), showing that the data captured the evolutionary alterations distinguishing resistant and sensitive cancer lineages.

To understand how the smoothing affected the genotype base calls, we examined each element (a specific site and cell in a matrix) of the read count matrices and calculated the percent that were altered by smoothing (Fig. 2B). Because smoothing depended on the depth of the coverage (the total number of reads), we discretized the elements into drop-out (0 reads, 66% of elements), low (1 read, 17% of elements), medium (2–4 reads, 13% of elements), and high (≥ 5 reads, 4% of elements) coverage. As expected, the genotypes from elements with low coverage were most likely to be altered by smoothing and were changed 63% of the time, in comparison to the ones with medium (34%) and high (30%) reads. The ability to call homozygous genotypes (either reference or alternate) improved with higher coverage. With low coverage, no heterozygous genotypes could be called without smoothing (since only one read was available), and ~ 60% of the homozygous genotypes were changed to heterozygous after smoothing. For elements with medium coverage, ~ 40% of the homozygous genotypes were changed; and for high coverage, ~ 10% were changed.

Finally, to determine if smoothing uncovered more sites associated with the resistance lineage, we examined the association of the 523 genomic sites with resistance status before and after smoothing. After smoothing, more sites showed genetic alterations that were associated with resistance/sensitive lineage status (Fig. 2C). These results indicated that smoothing enhanced the ability to identify sites linked to the evolutionary divergence of the resistant lineage.

### Phylogenies are not confounded by technical characteristics of scRNA-Seq

A major challenge in analyzing scRNA-Seq data has been the overall low coverage and high drop-out rates^19^. To assess the sensitivity of phylogenetic reconstruction to these characteristics of the data, we performed simulation studies using the CAMA-1 data set.

**Figure 3:**
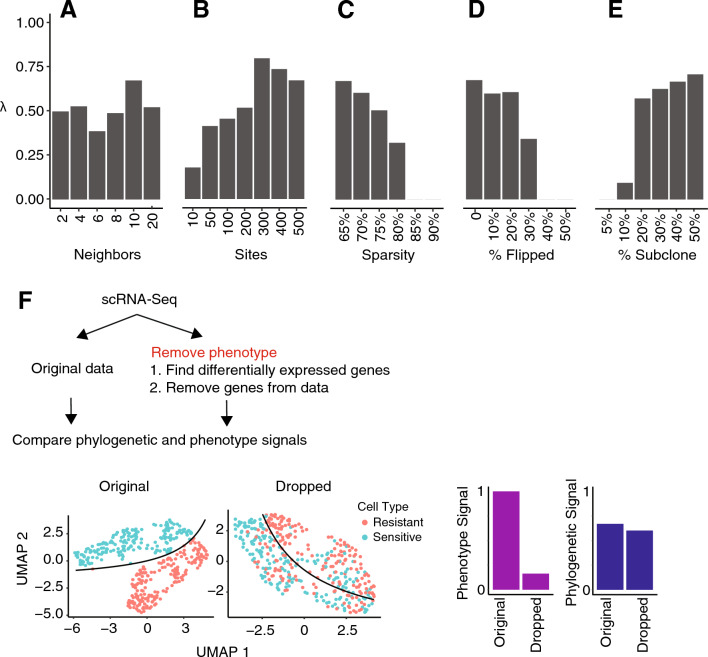
Impact of data quality and parameters on phylogeny. These barplots show the relationship between the phylogenetic signal λ and (**A**) number of neighbors, (**B**) number of sites, (**C**) sparsity, (**D**) percent of genotype flipped, and (**E**) subclone size as percent of all cells. (**F**) The graphic on the top shows the strategy we use to determine whether the phylogenies are confounded by gene expression patterns. The UMAP plots show the resistant and sensitive cells before (left) and after (right) dropping out 1948 genes associated with resistance. The bar plots represent the resistance signal identified from phenotype (left) and genotype (right) before and after dropping out the resistance-associated genes.

First, we tested the robustness of the inference to the number of neighbors, *K*, used in the smoothing algorithm (Fig. 3A). We generated phylogenies after varying *K* from two to 20 neighbors, and scored the association with resistance status using Pagel’s $\lambda ,$ a measure of phylogenetic signal^40^. We found that λ was not dependent on *K*, indicating that the phylogeny was relatively robust to the number of neighbors in this data set.

Next, we tested how the number of selected sites impacted the phylogenies (Fig. 3B). We reconstructed cancer phylogenies after selecting different numbers of genomic sites (10–500) with the highest average coverage. The phylogenetic signal increased as the number of sites increased up to 300 sites, and then began to decrease slowly, potentially due to the addition of noisier data. This suggested, in principle, that more sites should be favored over fewer, although too many low-quality sites can be detrimental. It is known that the number of sites required will increase with the number of evolutionary relationships to be inferred^41^. It is likely that the ideal number of sites depends on the mutational process that is driving the evolution of the cells, and thus may vary from data set to data set. The convergence of the evolutionary model onto a robust phylogenetic topology across MCMC iterations (post burn-in) is an indicator that the number of sites used is sufficient to resolve the evolutionary relatedness of the cells.

We also examined how the rate of drop-out affected the phylogenetic signal by taking the unaltered count matrix (with 65% missing values, which we called the *sparsity* of the matrix), and then setting random elements to 0 to increase the sparsity up to 90% (Fig. 3C). This experiment revealed that the phylogenetic signal decreased with the addition of sparsity, rapidly dropping after 75%. No association of phylogeny with resistance status was seen beyond 85% data sparsity.

Another potential source of noise was the incorrect genotype calls due to random sequencing artifacts or limitations of the imputation algorithm. To test whether these types of errors impacted phylogenetic inference, we simulated additional errors by randomly changing the genotypes of up to 50% of the elements (Fig. 3D). Up to 20% of the genotypes could be randomized with only a moderate decrease in the phylogenetic signal, but performance dropped drastically with more severe noise.

Next, we tested how well the algorithm could detect low frequency subclones. For this analysis, we constructed data sets consisting of the sensitive CAMA-1 cells, and then spiked in different proportions of resistant cells so that the resistant cell frequencies ranged from 5 to 50%, while maintaining a constant total cell count (Fig. 3E). To accommodate small subclones, we modified the filtering criteria and removed sites if over 95%, rather than the default of 90%, of the cells had the same base (see “Methods”). From analysis of the phylogenies, we found that subclones comprising at least 20% of the cells could be clearly detected, while smaller ones were difficult to detect.

Finally, we tested whether the gene expression patterns affected the phylogenies. Because genotypes could only be assessed (without imputation) from genes that were expressed, it was possible that the expression patterns may have confounded the structure of the phylogenies such that the clades reflected gene expression patterns rather than genotypes. Previously, we reported that resistant CAMA-1 cells had altered gene expression patterns^39^ that we confirmed here (Fig. 3F). We then eliminated the genes with differential expression between the resistant and sensitive lineages (dropping 1948 differentially expressed genes with log fold change > 0.05 and detected in > 5% of cells) and confirmed by UMAP that gene expression differences were substantially reduced. We then generated phylogenies from both the original and altered data sets. After removing the differentially expressed genes, we observed a dramatic decrease in the phenotypic distinction between the resistant and sensitive cells as quantified by the phenotypic signal, although it is possible there remains latent differences that are not detectable by differential expression analysis or UMAP visualization (Fig. 3F). However, the phylogenetic signal remained comparable, showing that the evolutionary divergence of resistant and sensitive cells could be identified from mutational changes, even if there were no differences in the gene expression patterns.

To summarize these results, we found that the single cell phylogenies reconstructed from scRNA-seq data were relatively robust to how our algorithm was applied, but could be affected by the quality of the data. We made several observations that could be used to guide applications of this algorithm to other data sets. First, we found that the phylogenies were relatively insensitive to the number of neighbors K, at least in this data set. Second, more sites (≥ 300) and less sparsity (less missingness: upper limit ~ 75%) are helpful in generating phylogenies. The algorithm itself is relatively robust to noise, tolerating up to ~ 20% of incorrect base calls. Further, we showed that the phylogenies were not confounded by the gene expression patterns. Finally, based on simulation studies, we saw that the algorithm could readily detect larger subclones comprising at least 20% of the cells, although smaller ones down to 10% were achievable. This suggests that the number of high-quality cells needed for the phylogeny must be 5× the expected size of the lineage of interest, after filtering.

### PhylinSic can reconstruct phylogenies across a range of biological conditions

Thus far, the results showed that our algorithm could recover the genetic differences underlying acquired drug resistance in breast cancer cells. To determine whether the method was generalizable and could recover evolutionary relationships produced by other evolutionary pressures, we tested it in a range of conditions seen in five public scRNA-Seq data sets where the subclonal structures were determined by independent data sources, including single cell DNA-sequencing or bulk whole genome sequencing (Table 1). Each data set contained monophyletic cells that could be grouped into subpopulations with distinct genetics. We reconstructed the phylogenies of cells in each data set and compared them against their previously established subclonal structure. Then, we measured the association between the phylogenies and the subclones using the phylogenetic signal.

**Table 1:** Gold standard data sets.

Data set	Description	Sequencing platform	Evidence of subclone	Cells	Sites	Sparsity (before filter) (%)	Sparsity (after filter) (%)	Reference
CAMA1	ER+/HER2− CAMA-1 breast cancer cells	10x 3′	Whole exome	400	213,379	97	65	PMID: 37386030
N87	Gastric cancer cell line	10x 3′	scDNA-Seq	500	12,984	92	75	PMID: 32215369
ER+ BRCA	Breast tumors, before and after treatment	Fluidigm Full transcript	Whole genome, scRNA-Seq copy number	141	164,580	97	53	PMID: 29093439
MM16	Multiple myeloma tumors, before and after treatment	Fluidigm Full transcript	Whole exome, scRNA-Seq copy number	46	144,057	94	32	PMID: 31558476
MM34	Multiple myeloma, primary and metastasis	Fluidigm Full transcript	Whole exome, scRNA-Seq copy number	127	326,089	95	16	PMID: 31558476

**Figure 4:**
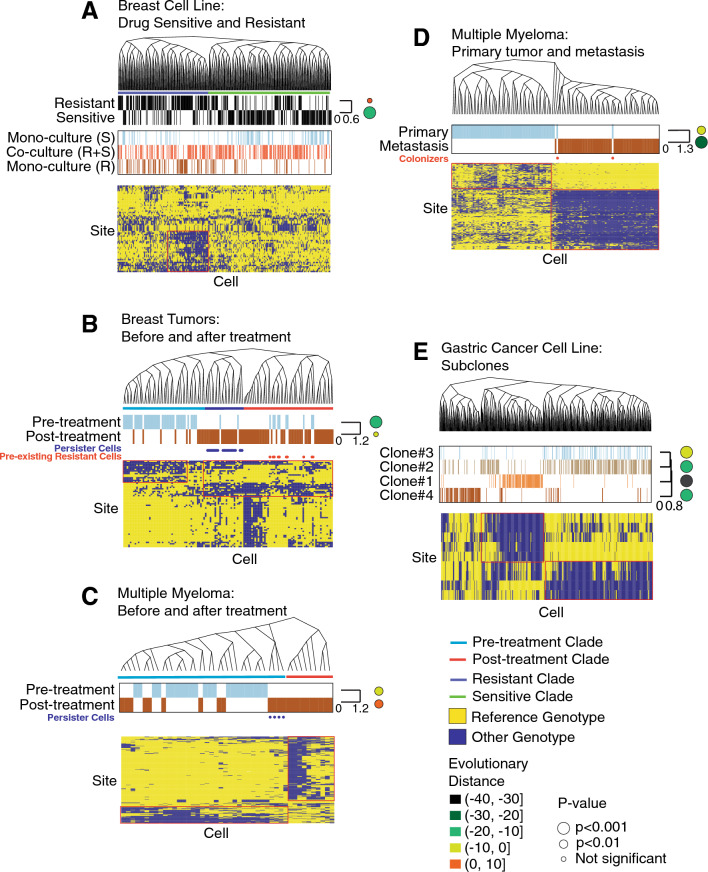
Phylogenetic reconstruction recovers previously identified subclonal lineages across a range of cancer settings. We have generated phylogenies from five scRNA-Seq data sets. The evolutionary distance for each sample condition is shown as the circles at the right of the heatmap. The color and size of the circles represent the evolutionary distance and statistical significance. The yellow (Reference) and blue (Other) heatmaps show the genotypes of the sites with phylogenetic signal K > 0.8 across cells in the phylogeny. If no sites achieved this threshold, we showed the 10 sites with highest K. (**A**) These cells were experimentally evolved from the parental CAMA-1 cell line to ribociclib sensitive (S) and resistant (R) cell lineages which were then grown in monoculture (alone) or mixed and grown in co-culture (R + S mixed in equal proportions). The cells induced to be drug resistant are shown as black lines in the *Resistance* bar. The genotypes of the sites with high association with the phylogeny are shown in the heatmap at the bottom. (**B**) This data set consists of tumor cells collected from ER+ breast cancer tumors before and after treatment. The color bars show the time point of each cell. The phylogeny is divided into clades of pre- (blue) and post-treatment (red) genotypes. Persister cells are delineated with dots. (**C**) This contains tumor cells from a multiple myeloma patient before and after chemotherapy treatment. The phylogeny is divided into clades of pre- (blue) and post-treatment (red) genotypes. Persister cells are indicated with dots. (**D**) This data set contains cancer cells collected from the primary or metastasis site from a multiple myeloma patient. (**E**) This data contains cells from the N87 gastric cancer cell line evolving under untreated conditions. Four subclonal lineages were previously reported to coexist.

We begin by investigating more deeply the CAMA-1 breast cancer cells from above^39^. In this data set, three samples were sequenced: one sample with resistant cells grown in monoculture, one with sensitive ones in monoculture, and one where the resistant and sensitive cells were co-cultured. After combining the cells from these three samples, we randomly selected 200 sensitive and 200 resistant cells and reconstructed their phylogeny. The phylogeny bifurcated into two major clades, where one was predominantly composed of resistant cells, and the other was mainly sensitive (phylogenetic signal $\lambda$ = 0.67, p = 2.8e^−23^) (Fig. 4A). There was no clear difference in the distribution of the monocultured or co-cultured cells, indicating that the phylogeny reflected genetic heterogeneity and was not confounded by environmental or batch sampling effects. The non-synonymous gene mutations used to generate this phylogeny, as well as the other ones presented in Fig. 4, are included in Table S1. As a baseline, we also generated this phylogeny with no genotype smoothing and observed no clear relationship between resistance and phylogenetic structure, confirming our previous results that smoothing leads to an improved ability to recover known biology (Fig. S1).

We then tested the ability of PhylinSic to infer genetic relationships from patient tumor data. In one patient derived tumor data set, we studied metastatic ER+ breast cancer tumor evolution^42^ with cells profiled from the same tumor both before and after treatment with chemotherapy (Fig. 4B). From this data set, we studied the evolutionary relationships of cancer cells in the tumor from the patient with the clearest evidence of subclone architecture (patient 3). In another data set, we analyzed tumors from patients with multiple myeloma (Fig. 4C)^43^. In both cases, the distribution of the pre- and post-treatment cells were associated with the phylogeny (breast $\lambda$ = 0.73, p = 1.5e^−17^, melanoma $\lambda$ = 1.1, p = 1.1e^−6^), indicating a shift in the genetics of the tumors after treatment. For the breast cancer tumors, a minority of the pre-treatment cells (23%) shared a genetic lineage with the post-treatment cells, indicating that some pre-treatment cells had a pre-existing resistant genotype, as we previously reported^42^. This pattern was not observed in the multiple myeloma tumors, although it was possible that too few cells were sequenced to detect this population.

To further characterize the evolutionary distinctness of pre- and post-treatment cells, we calculated the evolutionary diversity of cells across the phylogeny at each timepoint. We then assessed whether cells of each timepoint were phylogenetically clustered within specific branches of the evolutionary tree, using standard-effect-size mean pairwise distance (SES MPD). Negative evolutionary distances indicated phylogenetic clustering while positive distances indicated phylogenetic evenness across the tree. In both breast cancer and myeloma data sets, post-treatment cells were more evenly distributed across the phylogeny than pre-treatment cells, indicating that post-treatment cells were more genetically diverse despite the effects of drug selection.

In both breast and multiple myeloma tumors, we observed distinct pre- and post-treatment clades (Fig. 4B,C). Interestingly, in both tumors, a small set of cells that survived after treatment genetically resembled the pre-treatment genotype (inferred to be drug sensitive). That is, these *persister* cells were closely related genetically to the sensitive pre-treatment cells (they share a main lineage with the pre-treatment cells rather than post-treatment cells), but nevertheless survived after treatment (they are seen in the post-treatment sample) (Fig. 4B,C). One possible explanation for their resistance, despite genetic similarity to the sensitive cells, could be that the local tumor microenvironment provided resistance to these otherwise sensitive cells (e.g., by limiting drug penetration or providing growth stimuli). Additionally, pre-treatment we observed a small population of cells in the breast tumor that were genetically similar to the dominant post-treatment lineage (resistant cells), indicating the existence of resistant cancer genotypes within the heterogeneous tumor prior to treatment (Fig. 4B).

In addition to evolution over time, driven by drug treatment, we examined the evolutionary changes occurring at spatially distinct disease sites creating heterogeneity between primary and metastatic tumors from a multiple myeloma patient^43^. We found that the cells from the two tumors were nearly completely separated into distinct lineages (Fig. 4D). A small number of cells within the primary tumor belonged to the lineage that dominated in the metastasis, but the dominant lineage of the primary tumor was completely absent of metastatic cells. This result suggested that a single (or small number of related) genetic lineage(s) colonized the distant location and produced a distinct genetic population at the distant site.

Since PhylinSic could identify genetic changes resulting from drug treatment and metastasis, both of which were strong selective events, we finally tested whether it could detect differences resulting from more subtle genetic drift. To do this, we applied it to scRNA-Seq profiles of the N87 gastric cancer cell line that was cultured under conditions without any specific selective pressure^44^. We chose this cell line because, in the published study, it was the most deeply characterized. There, four subclones were identified by scDNA-Seq and scRNA-Seq. In the PhylinSic-derived phylogeny, distinct clades could be seen for Clone 1 ($\lambda$ = 1.1, p = 8.8e^−28^) and Clone 4 ($\lambda$ = 0.68, p = 1.8e^−79^), while we found some disagreement in the classification of Clones 2 ($\lambda$ = 0.5, p = 5.0e^−30^) and 3 ($\lambda$ = 0.34, p = 1.8e^−15^) which were more difficult to distinguish (Fig. 4E). The mutations that we identified supported the PhylinSic classification although some mutations detectable by scDNA-Seq may not have been observable in the scRNA-Seq.

Taken together, these results demonstrated that PhylinSic could reconstruct the phylogenetic relationships, enabling biological insight of cells across a range of biological settings (in vitro and in vivo throughout treatment or across metastatic sites) and sequencing platforms.

### Integrating genetic and phenotypic inference

Having seen the ability of PhylinSic to reconstruct the genetic relationships across single cells from scRNA-Seq data, we next sought to link these genotypes with the phenotypes of the cells, as represented by gene expression profiles. To do this, scRNA-Seq mutation profiles were first used for phylogeny construction, then phenotype associations were assessed based on the phenotypic similarity of cells within genetic lineages of the phylogeny. We analyzed the breast tumor data set (represented in Fig. 4B) to determine how phenotypes evolved in response to drug treatment.

**Figure 5:**
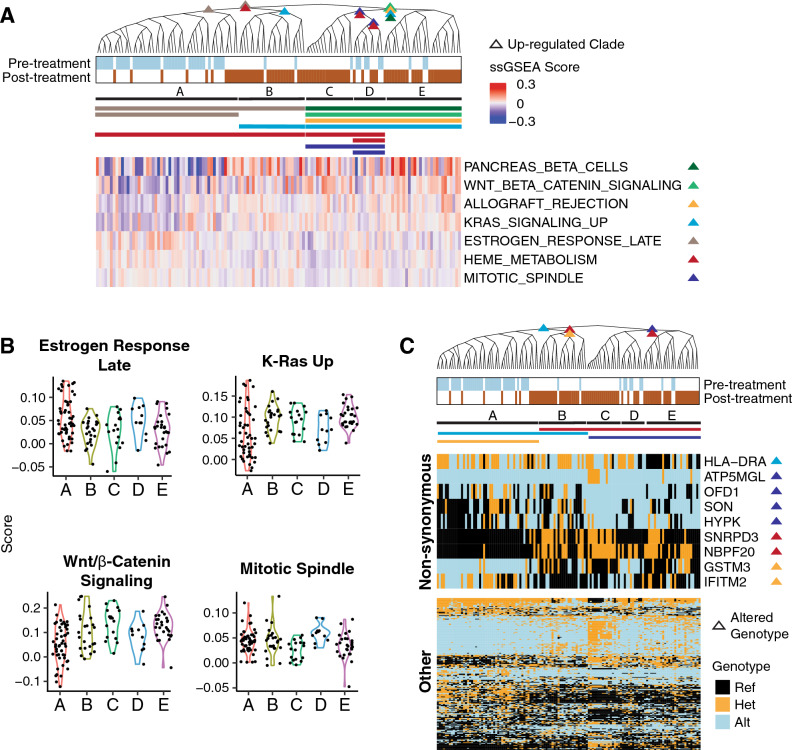
Associating the genotypes with phenotypes. (**A**) The phylogeny from the breast tumor data set in Fig. 4B is reproduced here with new annotations. The phylogeny is split into five clades (**A**–**E**). The clades associated with high pathway scores are marked with a colored triangle. The siblings of these clades have low pathway scores. The heatmap on the bottom shows the mean-centered ssGSEA scores for the Hallmark pathways significantly associated with the phylogeny based on phylogenetic signal (fdr < 0.05). Each pathway is labeled with colored triangle(s) that indicate the clade(s) with high pathway score. The phylogenetic associated clades are marked by the horizontal bars below the phylogeny. (**B**) The violin plots show the ssGSEA scores for the five clades. (**C**) This phylogeny and its clades are annotated similarly to those in figure A, except that gene mutations are associated with the phylogeny, rather than pathway scores. The heatmap on top shows genes with non-synonymous mutations that are correlated with the phylogeny. The bottom heatmap shows the remaining genes that are correlated with the phylogeny (fdr < 0.01).

To determine the phenotypic traits under evolutionary selection, we measured the phylogenetic signal λ for the Hallmarks ssGSEA pathways^45^. This revealed the phenotypes that were significantly dysregulated in each of the 5 major breast cancer clades (Fig. 5A: clades A–E). This included pathways previously linked with breast cancer including Wnt/β-Catenin signaling (false discovery rate, fdr = 0.005), K-Ras (fdr = 0.001), estrogen response (fdr = 0.035), and mitotic spindle (fdr = 0.002) (Fig. 5A,B). Many of these pathways were upregulated in only one clade of the phylogeny, predicting the event that led to activation of the pathway. For instance, one clade composed almost entirely of pre-treatment cells (clade A in Fig. 5A) had a high estrogen response signature, which was low in the remaining clades of post-treatment cells (clades B–E). Since these tumor cells were initially ER+, this predicted that there was loss of estrogen dependency after treatment, which was driven by a genetic change. In contrast, β-catenin signaling, a pathway correlated with drug resistance in a number of contexts^46,47^, was activated in the post-treatment cells.

Finally, in this data set, we noticed a set of persister cells, noted above, with a sensitive genotype that were retained after treatment (clade B in Fig. 5A). While these cells were genotypically distinct from the other post-treatment cells, we find here that some aspects of their phenotypes were similar (Fig. 5B). Notably, they shared high K-Ras and β-catenin pathway scores with the other resistant cells. However, while the other resistant cells had a stronger phylogenetic association with β-catenin, the persisters had a phylogenetic association with K-Ras. These results show that while the post-treatment cells had high activities for both pathways, the genetics underlying those pathways were different across the cancer clades.

To determine whether any recognizable mutations were associated with these phenotypes, we examined the 1000 mutation sites that were used to construct the phylogeny and measured their phylogenetic signal at each internal node of the phylogenetic tree (Fig. 5C). We found 228 sites that were significantly associated with the phylogeny (fdr < 0.01). The vast majority occurred in non-coding regions and thus were not likely to be driver mutations but support the reconstructed evolutionary relationships. We focused on the nine non-synonymous mutations and found one, GSTM3 (NM_000849, p.V224I), which had a significantly different genotype in the post-treatment cells and is a known β-catenin target that has been linked to chemoresistance and other phenotypes in cancer^48–50^. While the phylogeny could not determine whether this was a driver mutation activating a β-catenin signature without follow-up functional studies, it nevertheless provided a novel model of the biology and revealed hypotheses to be tested.

## Discussion

We have developed a method, PhylinSic, to reconstruct evolutionary relationships within tumors at the single-cell resolution and to link genotype to phenotype using scRNA-Seq data. We demonstrated the potential that this has for generating biological insight. Using probabilistic approaches, we addressed the challenges inherent across sequencing platforms, such as low read coverage, drop-out, and biases in the gene expression patterns. The approach we developed to call bases could also be applied to other contexts requiring accurate base calls from scRNA-Seq, such as estimates of mutation burden or calling of tumor vs. normal cell types. Finally, we have shown that our method, PhylinSic, can reconstruct phylogenetic trees of tumor evolutionary history for a range of data sets, from cell lines to patient tumors, and across a range of scRNA sequencing platforms including 10X Genomics and Fluidigm, covering both 3′ sequencing and full transcript protocols that vary in the depth and breadth of the coverage of the transcripts^47^.

Importantly, we found that smoothing the base calls using a kNN strategy resulted in a greater ability to generate genotypes and phylogenies that associated with known biology. Using simulation studies, we characterized the sensitivity of the phylogenies to each of the parameters of the algorithm. Somewhat surprisingly, we found that the phylogenies were relatively insensitive to the choice of K, the number of neighbors to consult when estimating the genomic profile of each cell. In principle, K is dependent upon the expected size of the subclones of interest, the genetic diversity within and between lineages, and the quality of the data. It is possible that our data set, which harbored two large lineages (resistant vs sensitive cells) that were likely to exhibit significant genetic differences due to powerful selection under drug treatment and also collected from clean samples from cell culture (rather than patient tumors), represented a situation that was not well suited to detect the relationships between K and phylogenetic structure. Therefore, we still recommend tuning K for each data set, setting an initial value based on the expected size of the subclones of interest.

In this study, we have validated the phylogenies reconstructed by PhylinSic in external data sets where subclones were determined by bulk whole exome sequencing, bulk whole genome sequencing, scDNA-seq, and copy number from scRNA-Seq. We recommend that users also utilize multi-omics approaches to validate the broad structure of the phylogeny and the benefits of differing levels of smoothing of mutation profiles, for example through rough measures of phylogenetic composition obtained from bulk DNA-sequencing. When DNA data is unavailable, phylogenies can be compared against copy number profiles estimated from the gene expression. Secondly, internal validation can be done by comparing the structure of the phylogeny against the expected evolutionary relationships of the cells. For instance, in a tumor, we expect cancer and non-cancer cells to form distinct clades and verification of this can support the broad evolutionary relationships recovered. To examine finer resolution evolutionary relationships, we recommend assessing the consistency of the mutational changes observed in the unsmoothed data and their specificity to distinct cancer clades.

After developing the phylogenies, in the in-depth analysis of drug resistance (in a CDK4/6 inhibitor and chemotherapy setting), we saw evidence for pre-existing populations of resistant genotypes, even pre-treatment. Intriguingly, in both tumor drug treatment data sets, we saw two distinct lineages of cells that survived after treatment. This result suggested that independent resistance mechanisms may commonly evolve in distinct lineages, leading to multiclonal tumors. Further, the phenotypic data showed that the post-treatment breast tumor cells, regardless of the lineage, had activation in at least two signaling pathways: K-Ras and β-catenin. While all resistant lineages had increased activation of those pathways, the differing genetic backgrounds suggested that they acquired activation through different mechanisms. This possibility implies that inhibition of multiple (or all) distinct resistance mechanisms may be necessary to achieve a durable response and thus supports the hypothesis that cancer heterogeneity is a key driver of tumor progression.

Resolving a full single-cell phylogenetic history of tumor evolution, beyond identification of subclones, provided the genetic relatedness of each cell, quantified from evolutionary distances that reflected the underlying mutation rates and divergence times. This allowed principled evolutionary associations between genotype and phenotype that enabled us to distinguish the traits associated with evolution, and also determine whether those traits evolved independently through convergent evolution–a situation that may confound cancer treatment^51^.

However, there remain limitations in our approach that need to be addressed. First, while we have seen that the mutation pattern can reveal phylogenetic structure, it generally cannot identify the mutations that drive evolution. New lineages can potentially be created by single mutations that confer a strong selective advantage. Because scRNA-Seq profiling, especially a 3′ biased protocol, does not provide a comprehensive view of the mutations, the drivers may be missed. Next, phylogenetic models, in principle, can reveal the timings of historical evolutionary events that could be related to events in the life of the patient (e.g., drug selection, time of metastasis, immune surveillance). However, this requires a carefully calibrated clock model that can contend with potential differences in the evolutionary rate across cancer cell lineages, for instance, due to differing abilities to repair DNA damage. This could be addressable with a model that integrates temporal scRNA-Seq samples that track mutation profiles through progression and describes the mutational processes that drive the rate of mutational divergence of each lineage from healthy reference cells^52^. Indeed, our pipeline allows users to select from a set of tree prior models describing different assumptions of tumor population growth during treatment. This includes simple parametric tree priors reflecting constant population size or exponential growth and also highly parametric models such as the Bayesian skyline model to describe complex patterns of cancer population growth that more often occur across treatments. This would also reveal how differing rates or processes of evolution among different lineages can impact the malignancy of the cells. Another complication arises from the fact that tumors can undergo many selective pressures as they develop, for instance, from resource limitations, immune surveillance, or drug treatment, that impact the population of tumor cells. Appropriate modeling of the evolutionary response to these events will require longitudinal samples as well as an appropriate model of the changes in population size^53^. In any case, the lens of evolution reveals not only the life history of the tumors, but provides an understanding of the current genetic state of the tumors, and potentially vulnerabilities in their evolutionary trajectory.

## Methods

### Generating alignments

We started by generating a BAM file containing the aligned reads for each cell. For the 10X scRNA sequencing platform, we used the standard CellRanger pipeline, which generated a BAM file (*possorted.bam*) containing the alignments for all the cells in each sample. We demultiplexed this file based on the error-corrected, confirmed barcodes for each cell in the CB tag (ignoring the GEM well suffix) in the alignment lines. Low coverage cells with less than 10,000 total reads were discarded, ensuring high data quality and accelerating genotype calling.

### Calling genotypes

We next identified the genomic positions where nucleotide bases differed across cells. To do this, we combined the demultiplexed BAM files into a single pseudobulk sample comprised of monophyletic cells (i.e., cells from the same person). The variants from the pseudobulk sample were then called using the GATK RNA-Seq pipeline^54,55^. In short, we added read groups, split reads, recalibrated base quality scores, realigned indels, and applied HaplotypeCaller. We filtered for the variants that were supported by at least 20 reads (across all cells), with at least five variant reads making up at least 5% of the total reads. This resulted in a list of the genomic sites that varied across the cells in the sample, which were used as candidates for subclonal mutations.

Next, for each of the variant sites, we extracted the number of reference and alternate allele reads from the cell-specific BAM files, resulting in two parallel matrices. One contained the number of reference reads for each of the sites (rows) and cells (columns). The other contained the number of alternate allele reads.

To account for the noise in the nucleotide read call, we used the pair of reference and alternate read count matrices to model the reference and alternate allele read counts as a probabilistic distribution over the alternate (or variant) allele frequency. We used a beta-binomial distribution where the number of reference and alternate alleles were observed (i.e., the number of alternate alleles were successes in a Bernoulli trial).

$$\mathrm{Beta}\left(\alpha ,\beta \right)=\frac{{p}^{\alpha -1}{\left(1-p\right)}^{\beta -1}}{B\left(\alpha , \beta \right)} \;\;\mathrm{for} \;\;0\le p \le 1$$

where $p$ was the variant allele frequency, $\alpha$ − 1 was the number of alternate reads and $\beta$ − 1 was the number of reference reads.

Based on the distribution of alternate allele frequency, we called the genotypes for each site in every cell. We distinguished among three possible genotypes: homozygous for the reference allele, homozygous for the alternate allele, or heterozygous. While theoretically, a reference genotype should only have yielded reference reads and an alternate genotype should only have yielded alternate reads, in practice, technical noise led to erroneous reads. To account for noise in the data, we introduced a parameter $\theta$ and obtained the probability that the underlying genotype was homozygous reference by integrating over the probability density function from 0 to $\theta$ (homozygous reference) or 1 − $\theta$ to 1 for a homozygous alternate genotype, and the remaining probability was allocated to the heterozygous genotype. The value of $\theta$ depended on the amount of noise in the data (which was difficult to measure), and we used $\theta$ = 0.3. We assigned a genotype by choosing the one with the highest probability.

### Smoothing genotypes

Due to the low read depth frequently seen in scRNA-Seq, most genotype calls were supported by few reads and were therefore uncertain. To improve the reliability of the calls, we adopted a smoothing strategy where we borrowed information from similar cells. We used a kNN approach where we identified cells with similar mutation profiles, and then calculated the average of their probability distributions, which were determined by read depth.

To find the neighbors of each cell, we used a mutation similarity score between each cell and all other cells. To calculate this score, we started by estimating the probability distribution over the three genotypes for each cell and site using the beta-binomial distribution, as described above. Then, for each cell, we sampled from the distribution for each site to yield a randomly simulated profile of the genotypes. To score the similarity of the genotype profiles between two cells, we used a Jaccard index (the percent of sites with the same genotype). The final similarity score between a pair of cells was the average Jaccard index over 100 samplings.

Using the pairwise similarity scores of each cell, we chose the *K* nearest neighbors for each cell. We smoothed the probabilities for each genotype by:

$${\widehat{p}}_{i,j,g}=(1-\delta ){p}_{i,j,g}+\delta \frac{\sum_{k}{p}_{k,j,g}}{K}$$

where *p*_*i,j,g*_ was the probability that cell *i* and site *j* had genotype *g* (either reference, alternate, or heterozygous). Since *K* was the number of neighbors used for smoothing, it should be less than the size, in cells, of the smallest clade of interest. $\delta$ was the fraction of probability mass to assign to the neighbors, and higher $\delta$ resulted in more smoothing. By default, we used *K* = 10 and $\delta$ = *K*/(*K* + 1).

### Filtering sites

Using the reference and alternate allele count matrices, along with the smoothed genotype calls, we filtered for high quality sites. In general, we first removed sites that (1) had low overall coverage (cells with < 10 reads), (2) had the same base in more than 90% of cells, (3) were located outside chromosomes 1–22, and (4) were within five nucleotides of another site. Next, we smoothed the genotypes as described above. Finally, we kept the 1000 sites with the highest number of reads. We used more forgiving thresholds (removed sites where cells had less than five reads, and had the same base in more than 80% of cells) for the CAMA-1 data set (see below) because it had relatively poor coverage. For the breast cancer data set, we smoothed genotypes using five neighboring cells due to the low number of cells.

For all remaining sites, we annotated the genomic metadata (e.g. genomic region, gene affected, impact on protein translation, etc.) using Annovar^56^.

### Generating and analyzing phylogenies

We formatted the filtered genotype matrix as a FASTA file for phylogenetic analysis. Each cell was converted into a sequence record containing the bases at each site. At homozygous sites, we used the reference or alternate bases observed in the sequencing data. For heterozygous sites, we chose a base other than the reference or alternate arbitrarily.

To model the phylogenetic relatedness of cells, we used BEAST2 with a relaxed log-normal clock model (RLN), generalized time-reversible site model (GTR) and Yule tree priors. The babette R package was used to construct models and interface with BEAST2^57^. We ran each Markov chain Monte Carlo (MCMC) chain for at least 100 million iterations and added iterations if convergence was not seen in the predicted phylogenies as visualized by a densitree, or by a plateau in the posterior probability of the model. We also monitored the mutation probabilities and rates. We summarized the model as a maximum clade credibility (MCC) tree.

### Phylogenetic and phenotypic measurements

The heritability of cancer traits was measured by the phenotypic resemblance of closely related cells. To characterize the phenotype of the cancer cells, we performed ssGSEA (single-sample gene set enrichment analysis)^45,58^ to obtain signature scores of the Hallmark pathways from the Molecular Signatures Database^45,59^. The pathway scores were normalized using a zero-inflated negative binomial model to account for the zero inflated (dropout) and over-dispersed read count data^60^. For each of the 50 Hallmark ssGSEA pathways, we used Pagel’s λ measure of phylogenetic signal to quantify the association between the pathway scores and the phylogenetic structure. This measure of phylogenetic heritability was calculated using the phylosig function in the Phytools R package^61^.

We also defined the phenotype signal as the ability to distinguish evolutionarily divergent cancer genotypes (known resistant and sensitive lineages) based on their RNA expression profiles (without mutation profile information). We quantified this by performing a UMAP clustering of the transcriptomic profile of all cells and used the adjusted random index to measure the distinctness of the phenotype of the genotypes using the adjustedRandIndex function implemented in the R package GeometricMorphometricsMix.

### Evolutionary diversity

The evolutionary diversity within a group of cells was measured by their mean pairwise (cophenetic) distance (MPD) across the phylogeny. The difference in evolutionary diversity of multiple groups of cancer cells (e.g. pre- vs post-treatment samples) was measured by the standardized effect size (SES) of the MPD within each group of cells, and was calculated using the ses.mpd function in the R package picante^62^. This identified samples with significantly more or less evolutionary diversity than would be expected by chance by randomly sampling from the phylogeny using a bootstrap permutation test.

### Gene expression analyses

For the CAMA-1 validation dataset, we identified the genes that distinguished drug resistant and sensitive cells so that we could verify that phylogenetic reconstruction was still possible after removing this phylogenetic signal. The expression profiles of the CAMA-1 single cells were processed using Seurat^63^. The count matrix was normalized using the LogNormalize method and the 3000 most variable genes (selection.method = vst) were identified. Principal component analysis (PCA) of the variable genes provided 50 principal components and UMAP (uniform manifold approximation and projection) further reduced the data into 10 dimensions. Next, the cells were clustered with SNN (shared nearest neighbors) in 10 dimensions with resolution = 0.5. Finally, we identified genes with log fold change > 0.05 between resistant and sensitive cells and are expressed in > 5% of cells. We removed these differentially expressed genes and verified by visualization in a UMAP plot that this lenient fold change cutoff led to the elimination of the differences in the gene expression profiles of the resistant and sensitive CAMA-1 cells.

### Supplementary Information

Supplementary Figure S1.

Supplementary Table S1.

## Data Availability

No new data sets were created for this study. The data sets analyzed are available in the Gene Expression Omnibus under GSE193278 (CAMA1 data set), GSE142750 (N87 data set), GSE110499 (MM16 and MM34 data sets); and the European Genome-phenome Archive under EGAS00001002436 (ER + BRCA data set). PhylinSic is available as a Snakemake pipeline on GitHub: https://github.com/U54Bioinformatics/PhylinSic_Project/.
