# Supplementary material for: Phylogenetic inference from single-cell RNA-seq data
Source: Sci Rep. 2023 Aug 8;13:12854. doi: 10.1038/s41598-023-39995-6 (PMC10409753; doi:10.1038/s41598-023-39995-6)
Supplement: Supplementary file 1 — Supplementary Figure S1. [file 41598_2023_39995_MOESM1_ESM.pdf]

Figure S1

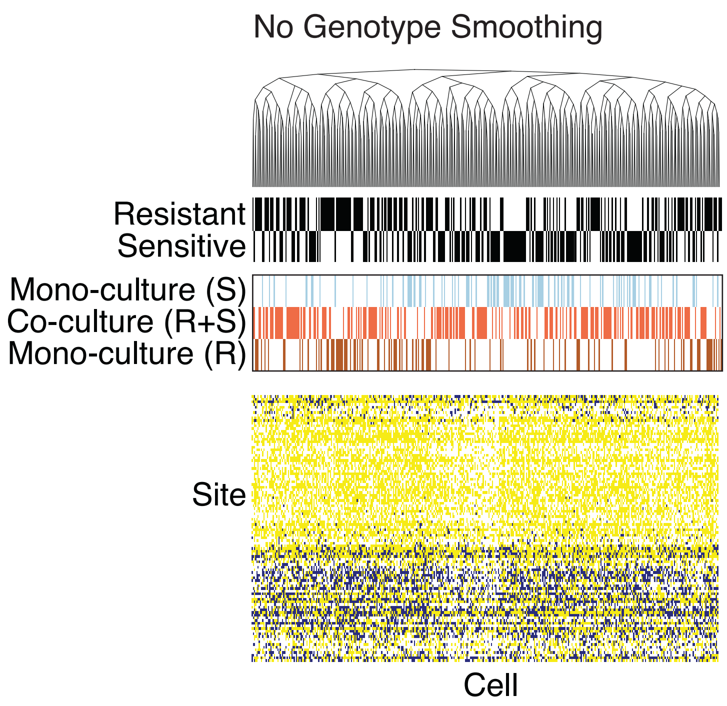

Figure S1. We generated a phylogeny from the CAMA-1 data set. Here, the parental cell line was evolved to acquire resistance to ribociclib. Sensitive and resistant cells were grown in either mono-culture (alone) or co-culture (both cells).

This phylogeny was generated using the same data and methods as the one in Figure 4A, except that no genotype smoothing was applied.
